# Radial free forearm flap versus pectoralis major pedicled flap for reconstruction in patients with tongue cancer: Assessment of quality of life

**DOI:** 10.4317/medoral.21274

**Published:** 2016-10-01

**Authors:** Wenlu Li, Peipei Zhang, Rui Li, Yiming Liu, Quancheng Kan

**Affiliations:** 1 MD, PHD. Department of Stomatolagy, the First Affiliated Hospital of Zhengzhou University, Zhengzhou, Henan 450052, China; 2 MD, DDS. Department of Stomatolagy, the First Affiliated Hospital of Zhengzhou University, Zhengzhou, Henan 450052, China; 3 MD, PHD. Department of Stomatolagy, the First Affiliated Hospital of Zhengzhou University, Zhengzhou, Henan 450052, China; 4 MD, PHD. Department of Stomatolagy, the First Affiliated Hospital of Zhengzhou University, Zhengzhou, Henan 450052, China; 5 MD, PHD. Department of Pharmacy, the First Affiliated Hospital of Zhengzhou University, Zhengzhou, Henan, 450052, China

## Abstract

**Background:**

This study investigated the quality of life of Chinese patients with tongue cancer who had undergone immediate flap reconstruction surgery. In addition, we compared 2 groups of patients: those who had received radial forearm free flap (RFFF) surgery and others who had received pectoralis major myocutaneous flap (PMMF) surgery.

**Material and Methods:**

Patients who received RFFF or PMMF reconstruction after primary tongue cancer treated with total and subtotal tongue resection were eligible for the current study. The patients’ demographic data, medical history, and quality of life scores (14-item Oral Health Impact Profile (OHIP-14) and the University of Washington Quality of Life (UW-QOL) questionnaires) were collected.

**Results:**

A total of 41 of 63 questionnaires were returned (65.08%). There were significant differences between the 2 groups in the gender (*p*< .05). Patients reconstructed with RFFF performed better in the shoulder domains, in addition to worse appearance domains.

**Conclusions:**

Using either RFFF or PMMF for reconstruction of defects after tongue cancer resection significantly influences a patient’s quality of life. Data from this study provide useful information for physicians and patients during their discussion of reconstruction modalities for tongue cancers.

**Key words:**Quality of life, radial forearm free flaps, pectoralis major myocutaneous flap, tongue cancer, oral function.

## Introduction

Soft-tissue reconstruction of the oral cavity, and in particular the tongue, that is the most critical factor in achieving a successful functional result. Malignant lesions occurring in the tongue are usually treated with primary surgical and/or radiotherapy of the head and neck region. Depending on the location and size of the tongue tumor, radical surgical treatment often affects all oral functions, such as speech, swallowing, chewing, oral rehabilitation, nutrition, and appearance (1). To maximize postoperative function, flap repair is currently the preferred method for reconstruction of defects after major surgery. For reconstruction of soft tissue defects, radial forearm free flaps and pectoralis major myocutaneous flap have proven to be very reliable and an average flap survival rate of 95% is usually achieved in experienced hands (2).

The pectoralis major myocutaneous flap (PMMF), based on the thoracoacromial artery, was described in 1979 by Ariyan (3). PMMF is well established as one of the most important reconstructive methods in major oral cancer surgery due to its simple technical aspects, versatility, and proximity to the oral cavity region (4). The role of the radial forearm flap in reconstruction is well established, and it is the principal form of reconstruction after radical excision of cancers of the oral cavity at the Royal Prince Alfred Hospital, Sydney (5).

Although the major intended outcome of oral cavity cancer surgery is still the survival of the patient, quality of life is now seen as an essential secondary outcome. Quality of life (QOL) has become an increasingly important outcome measure for patients being treated for many illnesses, as it reflects a patient’s general sense of well-being. It is by definition multidimensional and reflects the patient’s point of view (6). In addition, few studies have evaluated the differences in quality of life between patients with tongue cancers reconstructed with PMMF compared with those who underwent RFFF. Therefore, the aim of this study was to compare the differences between PMMF and RFFF for the reconstruction of the oral cavity defect in tongue cancer patients.

## Material and Methods

- Patients

The Institutional Ethics Committee of Zhengzhou University approved the study. Patients, who had had reconstructive surgery July 2005 and October 2013 in the Department of Stomatology, First Affiliated Hospital of Zhengzhou University, were eligible. Once patients had been diagnosed with tongue cancer they accepted that immediate reconstruction with different flaps was necessary.

Inclusion criteria for the study were: that the flap survived completely; the patient’s age was <65 years; patients had no previous or synchronous malignancies; patients had no cognitive impairment; QOL was assessed at least 12 months after reconstruction. Patients that suffered a recurrence of the disease were not excluded from the study.

In total, 63 patients (49 males, 14 females) met the inclusion criteria. Most patients completed the questionnaire when they returned to the hospital for their regular compliance review. The remaining patients received a formal letter explaining the study, an informed consent form, and 14-item Oral Health Impact Profile (OHIP-14) and the University of Washington Quality of Life (UW-QOL) questionnaires. Those patients who did not reply within 4 weeks received a reminder. Patients who were not able to fill in the questionnaires themselves (eg, due to dementia or language) were excluded from the study. Patient characteristics are summarized in  Table 1.

**Table 1:**
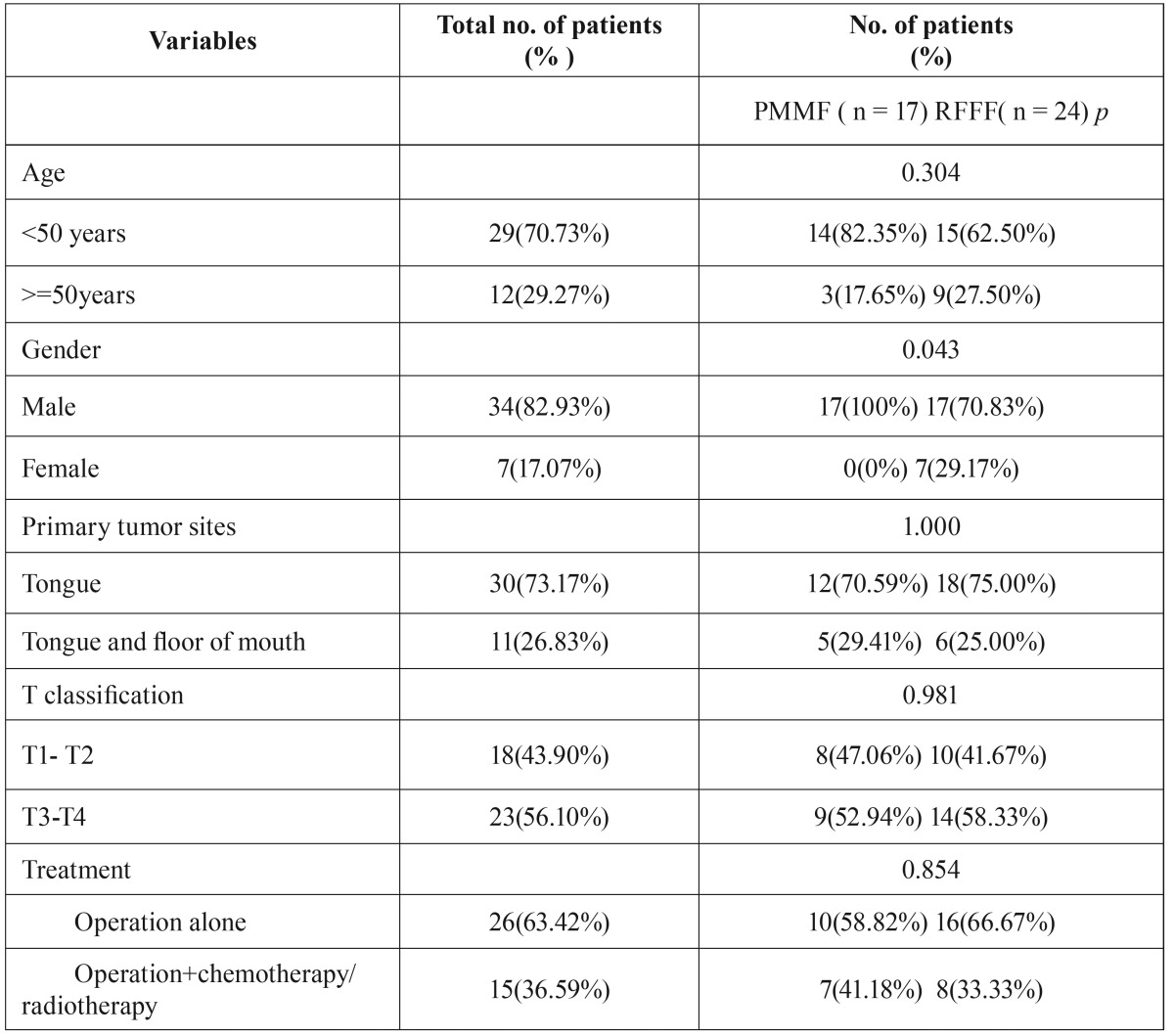
Clinical data analyses of tongue cancer patients who underwent PMMF or RFFF for reconstruction.

- Questionnaires and collection of data

The quality of life was assessed using the OHIP-14 (7)and UW-QOL (8) questionnaires. The OHIP-14 and the UW-QOL are two commonly used among patients with oral cancer. OHIP-14 consists of 14 items divided into 7 different domains: functional limitation, physical pain, psychological discomfort, physical disability, psychological disability,

social disability, and handicap. Each item is scored as: 0 = never; 1 = hardly ever; 2 = sometimes; 3 = fairly often; and 4 = very often. The domains are scored on a scale ranging from 0 (best) to 100 (worst). The higher the score, the poorer the patient’s state of health.

The most recently modified version of the University of Washington Quality of Life (UW-QOL) questionnaire was used in this study. This questionnaire evaluates the functional outcome of patients who underwent vascularized free forearm flap and free anterolateral thigh perforator flap reconstruction. The questionnaire is composed of 15 domains: 12 are disease-specific items (pain, appearance, activity, recreation, swallowing, chewing, speech, shoulder, taste, saliva, mood, and anxiety), and 3 are global questions. Each of the 12 included questions has 3-6 response options. The domains are scored on a scale ranging from 0 (worst) to 100 (best).

Besides the 15 questions, patients were asked to choose no more than 3 of the 12 disease-specific domains that had been the most important to them in the preceding 7 days. We scored the individual domains according to the UW-QOL guidelines.

- Statistical analysis

Data were recorded and analyzed with statistical soft- ware (SPSS 16.0; SPSS, Inc., Chicago, IL). Comparisons of nominal or ordinal variables between patients who underwent surgery with either the RFFF or PMMF were analyzed using a chi-squared test or Fisher’s exact test. The OHIP-14 and UW-QOL scores were compared for each domain using the nonparametric Mann-Whitney tests. The significance level was set to *p*< .05.

## Results

Of the 63 patients who were sent a questionnaire, 41 (65.09%) returned them completed. There were 8 questionnaires returned stating that the patients had changed their address and 7 that told us that the patients had died. Five patients refused to participate, two patient refused to sign the informed consent. Of the 41 patients who completed questionnaires, there were 34 men and 7 women, median age 53.5 (range 22-65); the postoperative follow-up period ranged from 13 months to 108 months. Tongue cancer in 30 cases, 11 cases of tongue and floor of mouth carcinoma. A total of 41 patients,8 cases of stage IV, 15 cases of stage III,18 patients in stage II. The type of 

flaps used were: 24 radial forearm free flaps (58.54%), 17 pectoralis major myocutaneous flap (41.46%). All 41 patients required soft tissue reconstructions alone. The average flap soft tissue area was 64 cm2. (Range 46-155cm2). 26 patients did not receive adjuvant radio-chemotherapy, 15 patients received either radio or combined radio-chemotherapy. ( Table 1)

There was no significant statistical difference between the PMMF and RFFF groups in age (*p*=0.304), primary tumor site (*p*=1.000), T-stage (*p*=0.981), and treatment (*p*=0.854). However, all of female patients received RFFF than did male patients (*P*<0.05). Furthermore, there were significant differences between the PMMF and RFFF groups in operation time (365 ± 48 vs 405 ± 107 min).

UW-QOL: In regard to the average scores of global QOL, no significant difference was found between the 2 groups (PMMF VS RFFF, 54.36±8.13vs55.14±9.24, *p*=0.965). There were also no significant differences between the 2 groups for the pain, activity, recreation, swallowing, chewing, taste, saliva, and anxiety domains. However, there were significant differences between the groups for the appearance (PMMF VS RFFF, 68.54±13.24vs57.47±11.44,*p*=0.001) and shoulder (PMMF VS RFFF, 54.65±11.24vs61.52±7.83, *p*=0.000) domains. When patients were asked to select their 3 most important domains, chewing was considered the most important, followed by saliva and taste. Pain domains were thought to be the least important ( Table 2).

**Table 2:**
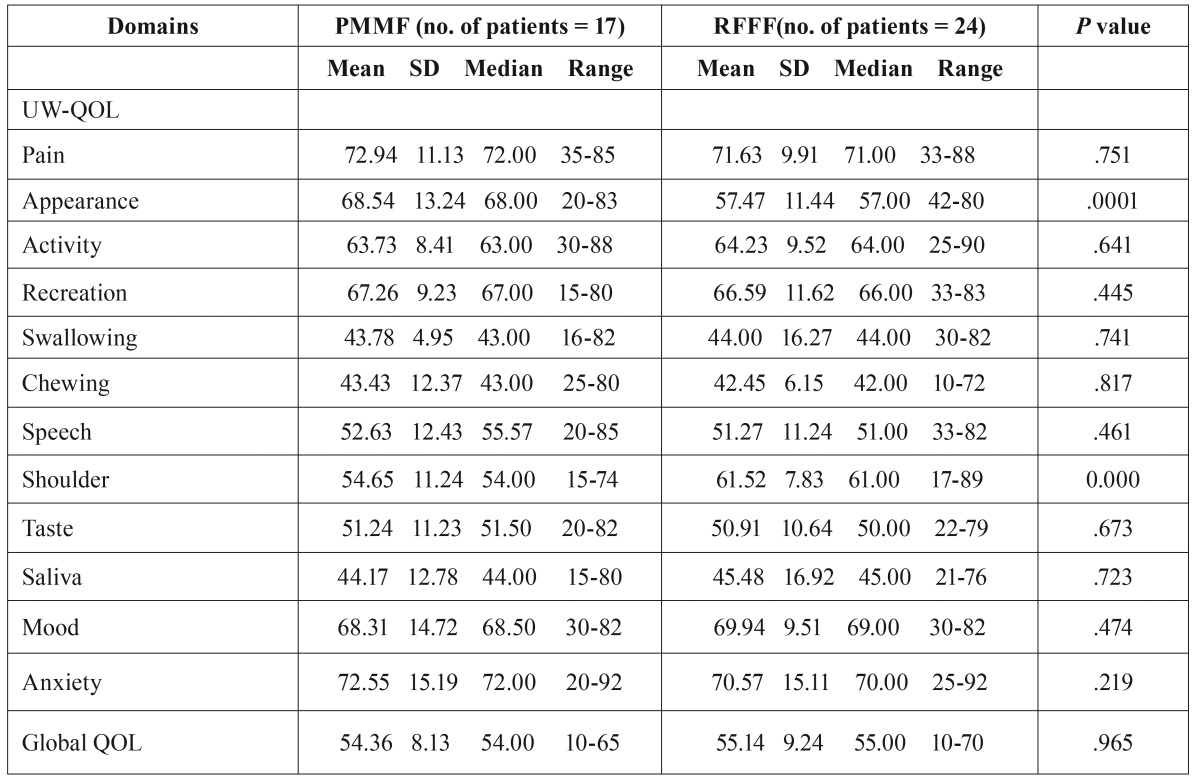
Means of scores of items and scales of UW-QOL questionnaire.

There was a significant difference between the Psychological discomfort (PMMF VS RFFF, 67.74±9.43 VS 61.53±8.56, *P*=0.000) and Social disability (PMMF VS RFFF, 45.13±9.37 VS 40.35±8.35, *P*=0.001) components of the OHIP-14 questionnaire between the 2 groups. The rest of the components did not show a significant difference ( Table 3).

**Table 3:**
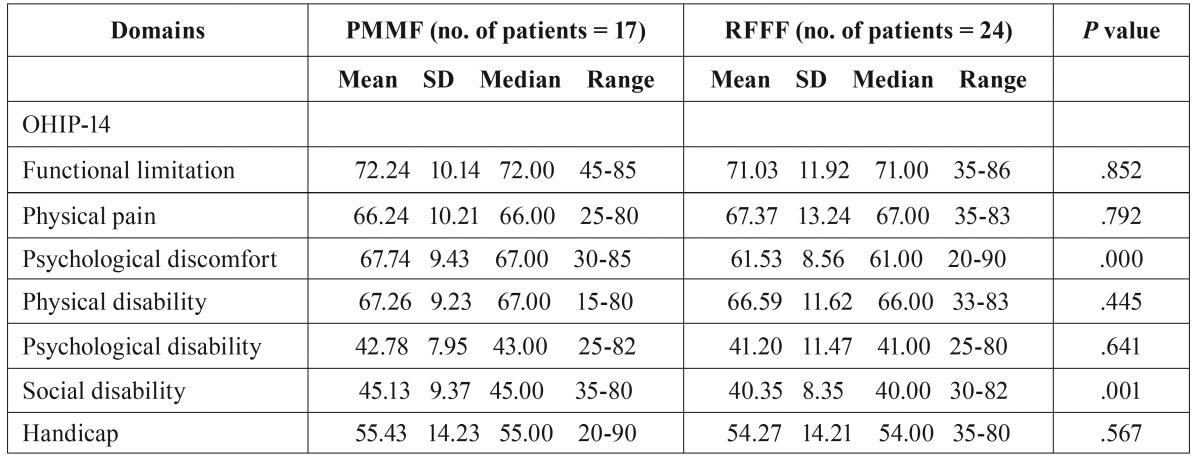
Means of scores of items and scales of OHIP-14 questionnaire.

In our survey, more than half of patients had a low level of education and only 9.7% of patients had a university degree. Only 13 patients (31.7%) had a stable income and the rest of the patients required funding from their family. In terms of living arrangements, 30 patients were married, 7 patients lived with their children, and 4 patients were divorced and lived alone.

## Discussion

Recording and reporting the postoperative effects of immediate repair with flaps is an important issue. This study is the first to directly compare QOL after the application of 2 different kinds of flaps using the 14-item Oral Health Impact Profile (OHIP-14) and the University of Washington Quality of Life (UW-QOL) questionnaires.

The tongue and floor of the mouth are the most common sites for primary cancers. The organs in the oral cavity, tongue is responsible for many different functions, such as the pickup and speech, swallowing, as well as in airway protection. The base of the tongue is more important for swallowing function, whereas the oral tongue is more important for speech and food manipulation.

Recording and reporting the malignant tumors of tongue after immediate repair with flaps is an important issue that can be approached in various ways. Proper reconstruction of tongue in the oral cavity may prevent complications, restore function, and improve quality of life and patient self-image. Providing basic tissue coverage without these considerations is no longer acceptable in modern-day practice (9).

Presently, it is generally acknowledged that free tissue transfer with micro-vascular anastomosis is the favored method for reconstruction after major head and neck cancer surgery (10). The free forearm flap procedure (Yang and colleagues) was chosen because the flap has a superficial location, it is anatomically constant, it has a long vascular pedicle, a thick diameter, and it is easy to cut, in addition to other advantages (11). This technique has been widely applied to patients requiring immediate repair of defects after oral cancer surgery. The PMMF provided reliable and ample vascularized soft tissue bulk with skin coverage for defects. The PMMF quickly became the flap of choice for primary reconstruction. Despite these benefits, regional pedicled flaps such as the PMMF were accompanied by a number of significant drawbacks including minimal pliability, a restrictive pedicle length, significant tissue bulk-limiting reconstruction of 3-dimensional defects, and long-term side effects of contracture with poor aesthetics (12). To the best of our knowledge, this study is the largest series to compare the differences between patients who have undergone PMMF and RFFF reconstruction after ablation of Tongue cancer.

QOL is an integral part of assessing the outcome of treatment for patients with oral cancer. As the oral cavity is responsible for many different functions, such as chewing of food, swallowing, production of saliva, speech, and breathing, and not least for interpersonal contacts such as kissing, a functional deficit leads to obvious changes in patients’ QoL (13).The expectation of the clinical outcome of reconstruction after operation for oral cancer is regarded as an important factor (14). The relatively large number of questionnaires specific for diseases of the oral cavity reflects that there is no ‘gold standard’. Our study used the OHIP-14 and UW-QOL questionnaires.

There was no significant statistical difference between the PMMF and RFFF groups in age (*p*=0.304), primary tumor site (*p*=1.000), T-stage (*p*=0.981), and treatment (*p*=0.854). However, all of female patients received RFFF than did male patients (*P*<0.05). Several previous studies found no significant difference in the gender distribution between RFFF and PMMF.However there was a higher proportion of female patients who underwent free flap reconstruction in the current study. This could be explanation might be presumed greater importance placed on cosmetic outcome (deformity of breast) among female patients resulting in a preference for RFFF in female.

Approximately 2 of 3 patients with head-and-neck cancer are treated with either chemotherapy or radiotherapy. After radiotherapy, some studies have suggested that weight, salivary function, and physical function were significantly reduced and that swallowing, coughing, and dry mouth symptoms increased (15). Previous studies have shown that adjuvant radiotherapy, compared with operation alone, results in the greatest functional deficit, resulting in persistent problems with disfigurement, chewing, and swallowing. Our work revealed other finding: 36.59 % patients in our study received postoperative radiotherapy or chemotherapy. However, there were no significant differences between the 2 groups.

We found that patients who received reconstruction with RFFF had a longer operative duration when compared with those who were reconstructed with PMMF (365 ± 48 vs 405 ± 107 min), which was a similar finding to that reported in previous studies (16). The need for microvascular anastomosis is may be the main reason for the longer duration of procedure.

The Chinese version of the OHIP-14, which has been translated and validated for use in Hong Kong, was used in this study (17). The OHIP-14 was designed to provide a comprehensive measure of the dysfunction, discomfort, and disability attributed to oral conditions. The OHIP-14 consists of 14 items organized into 7 subscales that assess how oral health can affect physical and social wellbeing. In addition the patient can complete it in 10min. In our study, there was a significant difference between the Psychological discomfort (PMMF VS RFFF, 67.74±9.43vs61.53±8.56,*p*=0.000) and Social disability (PMMF VS RFFF, 45.13±9.37vs40.35±8.35, *p*=0.001) components of the OHIP-14 questionnaire between the 2 groups. The rest of the components did not show a significant difference.

The questionnaire specifically designed for patients with head and neck cancer was better for demonstrating the changes in quality of life due to surgery. A relatively large number of specific questionnaires for the head and neck exist, indicating that there is no gold standard; therefore, many scholars use the UW-QOL questionnaire. The UW-QOL measure was chosen as the head and neck specific questionnaire because it is short and easy for patients to complete themselves, thus making it ideal in a busy outpatient setting. A remarkable finding was that there were significant differences between the groups for the appearance (PMMF VS RFFF, 68.54±13.24vs57.47±11.44,*p*=0.001) and shoulder (PMMF VS RFFF, 54.65±11.24vs61.52±7.83, *p*=0.000) domains. This is may be due to the donor site scar of RFFF which more likely to be exposed. However, the PMMF’ donor site scar was closed and hidden, allowing patients to easily accept donor site morbidity. We also found that the average score in the shoulder domain from the PMMF group was worse than that of the RFFF group. Some study showed that PMMF not only reduced the range of motion but also reduced the strength across more than one domain (16). This could explain why the average score in the shoulder domain in the PMMF group was worse than that of the RFFF group..

There were several limitations in our study. First, the sample size was small and may not have had sufficient power to find more valuable results.Second, this was not a randomized study. Selection bias inevitably existed.Some patients’ quality of life results may have been affected by chemotherapy or radiotherapy treatment that may last 3-6 months after completionof treatment.

## Conclusions

Quality of life is important when assessing the outcome of treatment for patients with tongue cancer. Using either RFFF or PMMF for reconstruction of oral defects after tongue cancer resection significantly influences a patient’s quality of life. Patients reconstructed with RFFF had better shoulder domain than patients who had undergone PMMF reconstruction, and this should be considered for future surgical planning. We hope that this study serves as a useful resource for physicians when they are selecting treatment modalities for tongue cancers.
